# Coconstructing CHAMP, an Artificial Intelligence Chatbot for Pediatric Infectious Symptoms Management: Protocol for a Multiphase Participatory Study

**DOI:** 10.2196/89852

**Published:** 2026-05-26

**Authors:** Jia Lin, Nikhil Jaiswal, Yuanchao Ma, Bertrand Lebouché, Sebastian Villanueva, Sofiane Achiche, David Lessard, Kim Engler, Simon Berthelot, David Buckeridge, Leo Anthony Celi, Isabelle Gagnon, Jocelyn Gravel, Laurie H Plotnick, Dan Poenaru, Marie-Pascale Pomey, Zoua M Vang, CHAMP Co-Construction Committee, Aida Berberovic, Esli Osmanlliu

**Affiliations:** 1 Centre for Outcomes Research and Evaluation, Research Institute of the McGill University Health Centre, Montreal, QC, Canada; 2 Department of Family Medicine, Faculty of Medicine and Health Sciences, McGill University, Montreal, QC, Canada; 3 Department of Pediatrics, Faculty of Medicine and Health Sciences, McGill University, Montreal, QC, Canada; 4 Chronic Viral Illness Service, Division of Infectious Diseases, Department of Medicine, McGill University Health Centre, Montreal, QC, Canada; 5 Department of Biomedical Engineering, Polytechnique Montréal, Montreal, QC, Canada; 6 Département de médecine de famille et de médecine d'urgence, Faculté de médecine, Université Laval, Québec City, QC, Canada; 7 Axe Santé des populations et Pratiques optimales en santé, Centre de recherche du CHU de Québec-Université Laval, Québec City, QC, Canada; 8 Department of Epidemiology, Biostatistics and Occupation Health, School of Population and Global Health, McGill University, Montreal, QC, Canada; 9 MIT Critical Data, Massachusetts Institute of Technology, Cambridge, MA, United States; 10 Laboratory for Computational Physiology, Harvard–MIT Division of Health Sciences and Technology, Cambridge, MA, United States; 11 Division of Pulmonary, Critical Care and Sleep Medicine, Beth Israel Deaconess Medical Center, Boston, MA, United States; 12 School of Physical and Occupational Therapy, Faculty of Medicine and Health Sciences, McGill University, Montreal, QC, Canada; 13 Department of Pediatrics, Division of Pediatric Emergency Medicine, Montreal Children’s Hospital, McGill University Health Center, 1001 Décarie Blvd, Montreal, QC, H4A 3J1, Canada, 1 514 412 4499; 14 Department of Pediatric Emergency Medicine, Centre Hospitalier Universitaire Sainte-Justine, Université de Montréal, Montreal, QC, Canada; 15 Department of Pediatric Surgery, Faculty of Medicine and Health Sciences, McGill University, Montreal, QC, Canada; 16 Harvey E Beardmore Division of Pediatric Surgery, Montreal Children’s Hospital, McGill University Health Centre, Montreal, QC, Canada; 17 Department of Management, Evaluation and Health Policy, School of Public Health, Université de Montréal, Montreal, QC, Canada; 18 Department of Family Medicine and Emergency Medicine, Faculty of Medicine, Université de Montréal, Montreal, QC, Canada; 19 School of Human Ecology, University of Wisconsin-Madison, Madison, WI, United States; 20 See Acknowledgments

**Keywords:** chatbot, artificial intelligence, large language models, pediatric infections, emergency medicine, self-management, patient engagement, participatory research, coconstruction, digital health

## Abstract

**Background:**

Acute infectious symptoms are a leading cause of pediatric emergency department visits in Canada, many of which are low acuity and could be safely managed at home. Artificial intelligence (AI) chatbots offer a promising avenue for delivering accessible, evidence-based guidance to support families in managing these symptoms.

**Objective:**

This study aims to adapt and coconstruct CHAMP (CHatbot to Assist the Management of Pediatric patients), an AI chatbot to support patients and families with acute pediatric infectious symptoms. CHAMP aims to deliver timely, tailored, and validated health information to support safe at-home self-management and informed care-seeking.

**Methods:**

This multiphase, mixed methods participatory study will be conducted at the Montreal Children’s Hospital in Montreal, Quebec, Canada. A coconstruction committee comprised of youth, parents, caregivers, and partners will be engaged as coresearchers. Eligible participants will include (1) youth aged 14‐17 years and (2) parents and caregivers of children aged 0‐17 years. The study comprises 5 phases. Phase 1 involves a qualitative needs assessment using focus groups with 20 participants to explore their informational needs, preferences, and concerns regarding pediatric infections and the use of AI chatbots. Phase 2 focuses on coconstructing and validating CHAMP’s knowledge database through 3‐5 workshops. Coresearchers will review pediatric clinical guidelines, map care questions and decision-making processes, and shape CHAMP’s conversational framework. Phase 3 consists of iterative prototyping and testing through 3‐5 workshops. Coresearchers will engage in prototyping and scenario testing, alongside preliminary usability and acceptability assessments. Phase 4 examines equity and accessibility through focus groups with 20 participants at risk of digital exclusion, as well as multilingual evaluation of an automated large language model–based translation layer. Phase 5 uses collaborative ethnography to explore the process of participatory coconstruction and its impact on CHAMP’s design.

**Results:**

Funding was secured in 2024, and Research Ethics Board approval was obtained in December 2024. As of December 2025, the coconstruction committee is being assembled, and Phase 1 recruitment is underway.

**Conclusions:**

This study will produce a functioning CHAMP prototype grounded in participatory, equitable, and responsible pediatric AI development. Findings will inform usability testing and an implementation-effectiveness evaluation, contributing to best practices for pediatric-centered AI health tools. By providing timely, tailored, and validated health information on acute infections, CHAMP may support safe at-home self-management, reduce preventable emergency department visits, ensure at-risk children are directed to appropriate care, and improve patient and family health care experiences.

## Introduction

### Background

Every year, nearly 2 million children visit Canadian emergency departments (EDs) [1], with acute infectious symptoms (eg, fever, cough, and vomiting) among the leading causes [2]. Many of these encounters are “preventable” ED visits: low acuity conditions that could be safely managed at home or in primary care [3]. National data indicate that 26% of all ED visits among children aged 2-9 years between 2023 and 2024 were for conditions manageable at home or in primary care, the highest proportion of any age group [4]. At the Montreal Children’s Hospital in Quebec, ED consultations for minor health problems can account for over 50% of all visits, particularly during peak viral seasons [5]. Preventable ED visits represent low-value health care encounters, contributing to fragmented care, negative family experiences, excess health care costs, and system-level strain [6-8].

Timely access to reliable health information can enable families to successfully self-manage many acute infectious symptoms at home, reducing unnecessary ED use and improving parent and caregiver confidence [9]. However, families often report difficulty reaching health care professionals or identifying trustworthy online guidance, especially amid the growing prevalence of disinformation and misinformation (ie, false or misleading information with or without the intent to deceive) [10,11]. There is a need for scalable, accessible, and evidence-based tools to help families navigate common, nonemergent pediatric infections outside ED settings while maintaining safety and equity in access to care.

Many pediatric infectious symptoms follow repetitive and predictable care patterns (eg, standardized symptom monitoring, hydration guidance, and return-to-care criteria) [12], which create a compelling opportunity for automated digital innovation, particularly through chatbot technology. Chatbots are software applications that simulate human-like conversation through text or speech [13]. Their use in health care is growing [13-18], accelerated by advances in large language models (LLMs), which are artificial intelligence (AI) systems designed to understand, generate, and interact with human language [19]. AI chatbots can deliver interactive, personalized, and evidence-based health information, which may improve pediatric knowledge mobilization among families [14]. By providing accessible and reliable health information, chatbots can bridge knowledge gaps, clarify home-management strategies, highlight red flag symptoms, and guide care-seeking options [14]. These capabilities can support safe self-management, informed decision-making, and optimize clinical pathways, reducing preventable ED visits while ensuring that at-risk children are directed to appropriate care.

Nevertheless, concerns remain about the effectiveness, safety, and equity implications of AI chatbots in health care. AI chatbots may perpetuate biases and exacerbate inequities if not designed and implemented carefully with diverse populations in mind [20,21]. Many existing systems are privately developed, general-purpose models such as ChatGPT (OpenAI), trained on broad internet data not curated for clinical reliability [22,23]. As a result, they can generate inaccurate or nonsensical outputs delivered with apparent confidence and perceived legitimacy, creating risks for misinformation, misplaced trust, and compromised safety [24,25]. The “black box” nature of LLMs further constrains transparency, complicates safety assurance, and impedes alignment with local clinical guidelines [26].

A growing body of literature emphasizes the importance of participatory development and meaningful patient and public engagement to support equitable AI design and implementation in health care [20,21]. However, many AI chatbots have been developed with minimal input from patients, families, or communities, and early-stage patient or public engagement remains rare [20,27,28]. This gap is even more pronounced in pediatrics, where children and youth are seldom included in the development or evaluation of clinical AI tools [29,30]. Moreover, few AI chatbots have been optimized for pediatric contexts, contributing to potential risks of age-related algorithmic bias and performance gaps [30-32]. Ensuring representation of diverse voices, particularly underserved groups, is critical in designing inclusive AI chatbots that can mitigate, rather than reinforce, health inequities [20,21,33,34]. Yet, without in-depth attention to how engagement unfolds in practice, patient and public input can risk becoming tokenistic rather than substantive [35]. Accordingly, little is known about how participatory processes are enacted in practice, negotiated among patients and partners, or constrained by technical, institutional, and ethical considerations during AI development. Given these limitations, an urgent need exists for evidence-based, context-sensitive AI health chatbots, designed through inclusive, transparent, participatory methods, particularly for pediatric care.

In 2020, members of our team (YM, SA, and BL) co-developed MARVIN (Minimal AntiRetroViral INterference), a bilingual (English and French) AI health chatbot with patients and partners to support antiretroviral therapy self-management for people living with HIV [36,37]. MARVIN’s conversational features addressed three key user needs: (1) guidance on antiretroviral therapy, (2) travel-related medication management, and (3) general HIV information [36,37]. Built using the Rasa open-source framework (Rasa Technologies Inc) [38], its architecture integrated multiple algorithms (ie, intent classification, entity extraction, a FallbackClassifier, memorization policy, and rule-based policy) operating across 3 modules: natural language understanding, dialogue management, and response selection [36,37]. A recent pilot trial demonstrated MARVIN’s usability and acceptability for HIV self-management [37], suggesting its potential for adaptation to other clinical domains and populations, including pediatrics [36].

### Aims and Objectives

In partnership with youth, parents, caregivers, and partners, this study aims to adapt the MARVIN architecture to develop CHAMP (CHatbot to Assist the Management of Pediatric patients), an AI chatbot designed to address the unique needs of pediatric populations with acute infectious symptoms. CHAMP is not intended to provide diagnostic services; rather, it will deliver timely, tailored, and validated health information to support safe at-home symptom management and informed care-seeking. CHAMP’s adaptation will involve reconfiguring MARVIN’s conversational architecture, knowledge database, and dialogue logic to reflect pediatric-specific clinical guidelines. CHAMP’s content will also be delivered in plain language and grounded in the real-world decision-making contexts of patients and families.

These aims will be achieved through the following objectives:

1. Identify the informational needs, preferences, and concerns of youth, parents, and caregivers regarding pediatric infections and chatbot use.
2. Coconstruct and validate a user-informed chatbot knowledge database, grounded in patient and family priorities.
3. Iteratively design and refine the CHAMP interface through participatory coconstruction.
4. Identify and address barriers to CHAMP use among pediatric populations at risk of digital exclusion.
5. Explore the longitudinal process of participatory coconstruction and its impact on CHAMP’s development.

## Methods

### Adaptive Trial Approach: From MARVIN to CHAMP

CHAMP’s development will follow the MARVIN master protocol, which outlines a flexible and adaptive trial approach for translating the MARVIN chatbot infrastructure to diverse clinical populations [36]. This study serves as a working example of this adaptive protocol. While future phases will address usability testing and implementation-effectiveness evaluation, this study focuses on the early development of CHAMP. Grounding CHAMP in the MARVIN protocol ensures methodological consistency while remaining responsive to the specific needs of pediatric patients and families.

The study is led by an interdisciplinary team comprising researchers, health care providers, engineers, patient and family partners, community collaborators, and students, ensuring diverse expertise across clinical, technical, and lived experience domains. This protocol is registered on ClinicalTrials.gov (NCT05789901). It follows the SPIRIT-Al (Standard Protocol Items: Recommendations for Interventional Trials–Artificial Intelligence) [39] and GRIPP2 (Guidance for Reporting Involvement of Patients and the Public) guidelines [40].

### CHAMP Technical Overview

**Figure 1.**
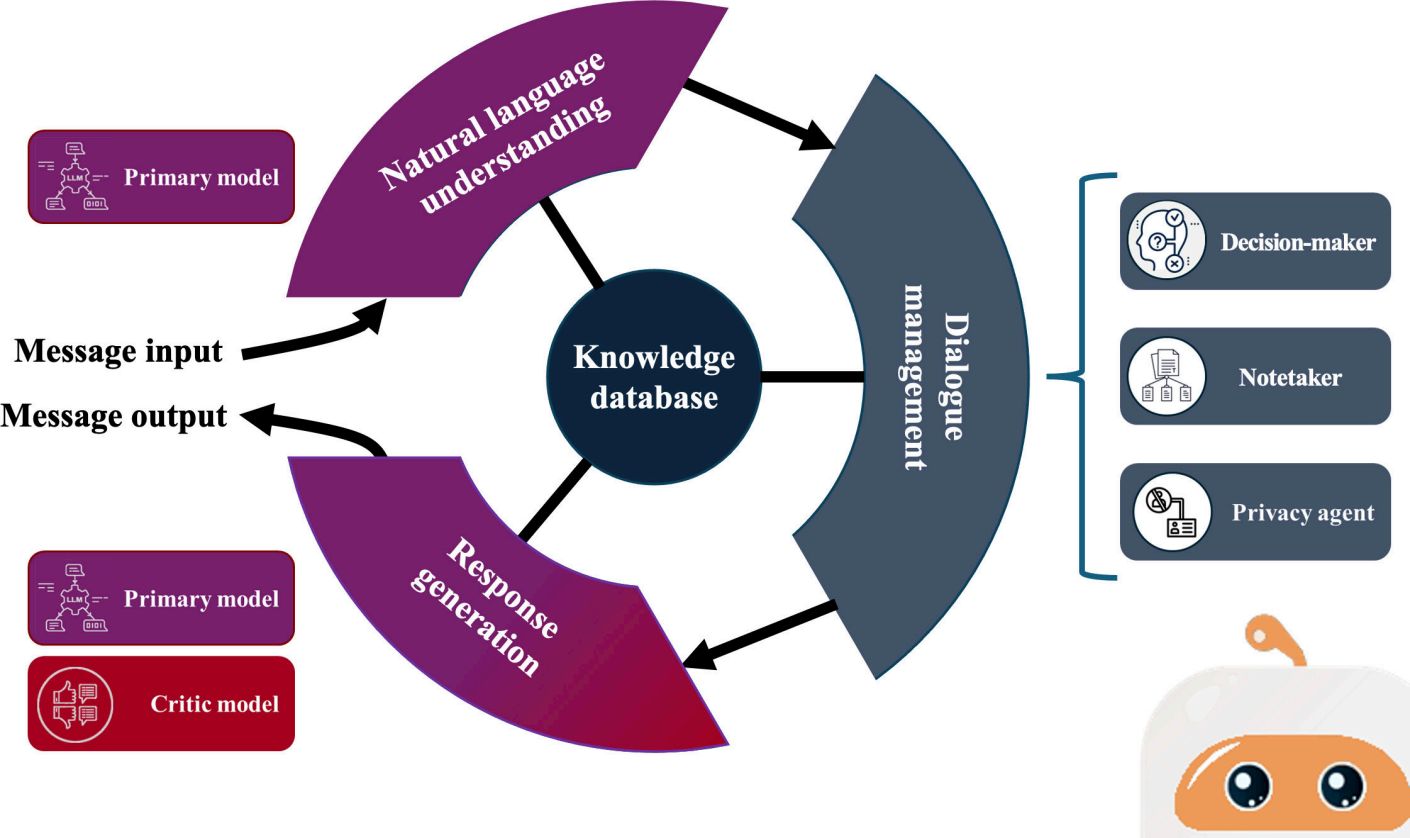
CHAMP (CHatbot to Assist the Management of Pediatric patients) multiagent architecture.

Like MARVIN, CHAMP will enable human-like conversations with users. We will use an adapted version of the MARVIN infrastructure, while leveraging a novel multiagent architecture (Figure 1). CHAMP will adopt open-sourced LLMs, primarily GPT-OSS-20B (OpenAI) [41], as the foundation of this architecture. In this approach, an ensemble of different LLMs will interact with a rigorously validated knowledge database tailored to pediatric acute infectious conditions and clinical guidelines [30]. To initiate this database, the research team has integrated high-quality, open-access, validated pediatric health resources from 2 Québec-based knowledge mobilization organizations: Institut National de Santé Publique du Québec [42] and Naître et Grandir [43].

CHAMP will accept textual user input, all of which will be processed directly by the LLM without human involvement. User queries will be managed through a retrieval-augmented generation workflow that links inputs to the validated knowledge database and restricts responses to information within its scope. When inputs are ambiguous, poor-quality, or outside this domain, CHAMP will return a standardized clarification message (eg, “I don’t have information on that topic”). To ensure accessibility, CHAMP will be designed to interpret everyday language at an approximate grade 6 reading level, and no prior clinical or technical expertise will be required from users.

### Study Design

This study is grounded in pragmatic assumptions typical of problem-centered and implementation-focused health research [44,45]. It privileges actionable knowledge and the real-world contexts in which digital health tools are developed, drawing on multiple methods of inquiry to best address the research problem [46].

**Figure 2.**
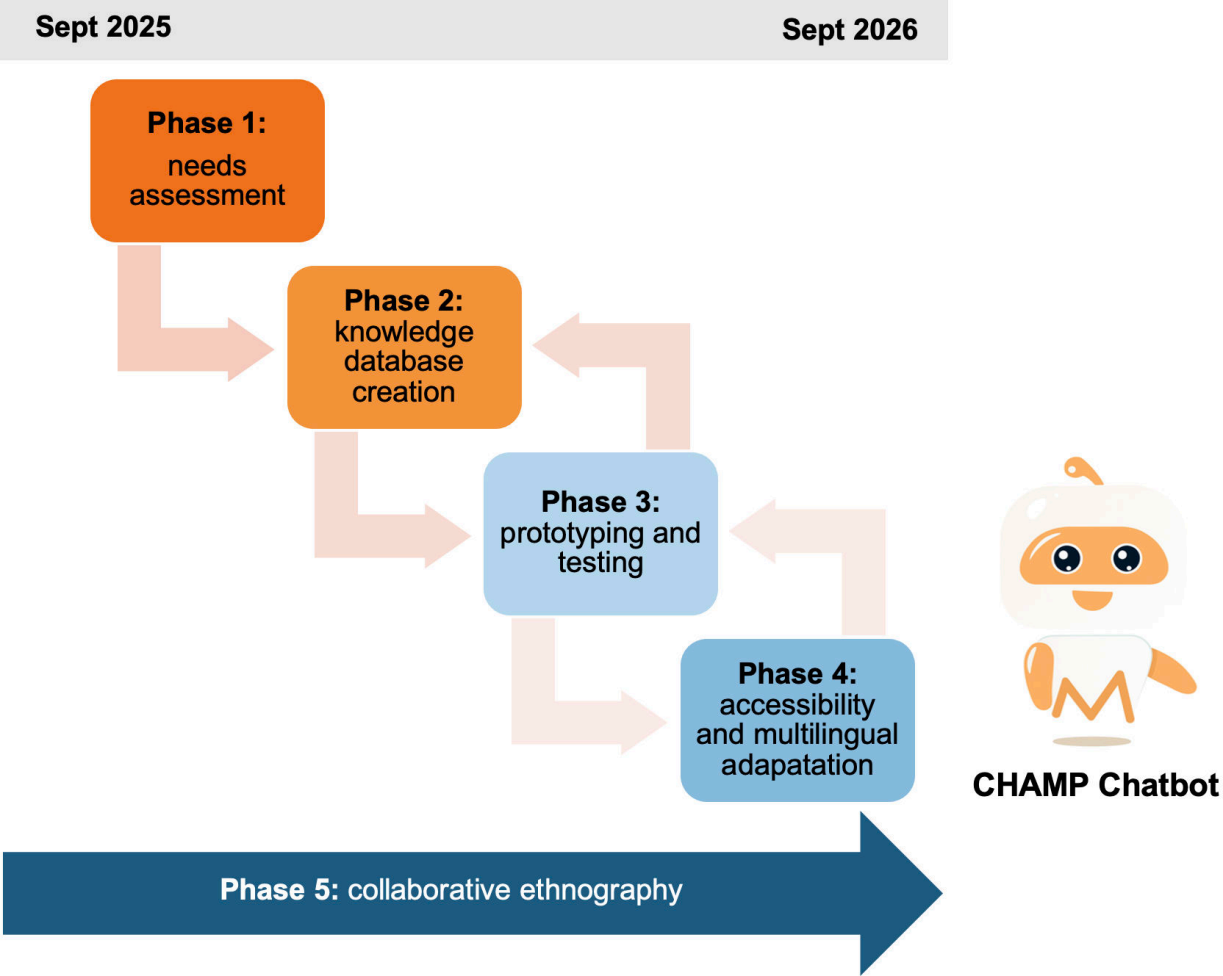
Multiphased study process.

We will use a multiphase, mixed methods, participatory approach to guide CHAMP’s development (Figure 2) [44,46,47]. This approach allows iterative incorporation of user perspectives (eg, needs assessment and coconstruction workshops), real-time adaptation of chatbot features and knowledge content, and risk mitigation by piloting features before full-scale implementation. Combining qualitative and quantitative methods provides a comprehensive understanding of the nature and underlying reasons for research outcomes [46]. Triangulation across methods strengthens the validity of findings to support nuanced, contextually grounded interpretations [46].

A participatory approach is central to this study. Within this paradigm, people with lived experience are engaged as active partners across the research lifecycle rather than as study participants [47,48]. Engaging youth, parents, and caregivers aims to address the ethical concerns of power asymmetry in digital health research and the need for inclusive AI development [48]. In CHAMP, a participatory approach frames the entire study, and youth and family partners are engaged from study inception to dissemination to enhance the relevance, trust, and acceptability of CHAMP [47].

### Patient and Public Involvement

Within a participatory paradigm, we will follow the Montreal model for patient engagement [47], embedding coconstruction as the central collaborative process in both research and chatbot development. Coconstruction positions partners as active coresearchers in shaping CHAMP by jointly defining priorities, informing key decisions about content and clinical logic, and grounding the tool in lived experience.

Youth, parents, caregivers, and partners will form a coconstruction committee (12‐15 members). Two groups will be engaged as coresearchers: (1) established partners from study conception and (2) newly recruited participants from Phase 1 (needs assessment). Following Phase 1, interested participants will be invited to join the coconstruction committee, bringing together existing and newly engaged partners. Coresearchers will participate in iterative workshops (Phases 2 and 3) to inform CHAMP’s development and broader research activities, including: (1) codeveloping grant applications; (2) attending team meetings; (3) informing study design, execution, and interpretation; and (4) coproducing knowledge dissemination products. Engagement intensity may vary over time for different members of the coconstruction committee [49]. To promote retention, we will emphasize shared ownership, regular communication, and flexible involvement, which are expected to strengthen members’ sense of commitment and ownership of the research [49]. Coresearchers will receive honoraria in accordance with the Strategy for Patient-Oriented Research Evidence Alliance and CHILD-BRIGHT Network guidelines [50,51]. A core aim is to build sustainable partnerships that extend beyond the study’s duration.

### Setting and Participants

This study will be coordinated from the Montreal Children’s Hospital located in Montreal, Quebec, Canada. The Montreal Children’s Hospital ED sees over 70,000 annual visits and serves families with diverse linguistic and digital literacy backgrounds. Eligible study participants include (1) youth aged 14‐17 years and (2) parents and caregivers of children aged 0‐17 years. The primary inclusion criteria are as follows: (1) visited a Canadian pediatric ED in the past 2 years; (2) speak and read English, French, Arabic, Spanish, Italian, or Mandarin; and (3) can understand the study requirements and provide informed consent. The language consideration reflects the 6 most commonly spoken languages in Montreal [52]. Individuals will be excluded if they are affected by severe psychological or cognitive impairments or comorbidities that limit their functional capacity.

### Recruitment

Participants will be recruited through multiple channels, including the Montreal Children’s Hospital ED, community-based organizations, and professional associations. In the ED, a research assistant will collaborate with clinical staff to identify and approach eligible youth, parents, and caregivers through convenience sampling. We will also use purposive sampling through partnerships with community organizations to ensure involvement of families experiencing digital access barriers, including those facing socioeconomic disadvantage, precarious immigration status, or limited literacy skills. Our network of community collaborators includes PROMIS*,* an organization supporting recent immigrants in Montreal; Garage à Musique, a community social pediatrics center serving families with complex social needs; and Première Ressource, aide aux parents, which supports families across Quebec.

### Equity, Diversity, and Inclusion

To promote equitable participation, we will offer hybrid meeting formats (virtual and in-person) to support individuals living in remote areas or those for whom in-person attendance is challenging. Scheduling will consider diverse preferences, including individuals with nonstandard work schedules. Occasional asynchronous participation will also be supported through meeting recordings, emails, and phone interactions. We will offer live translation services and additional financial compensation for childcare responsibilities and transportation.

### Study Process: Coconstructing CHAMP

#### Overview

The study protocol follows an iterative, phased approach to develop CHAMP (refer to Table 1). Each phase aligns directly with each research objective.

**Table 1.** Study objectives and phases.

Phase	Objective	Participants or partners	Qualitative	Quantitative	Outcomes
Needs assessment	Identify needs, preferences, and concerns about pediatric infections, AI^a^, and chatbot use.	10 youths and 10 parents and caregivers overall	Thematic analysis of focus groups guided by the NASSS^b^ framework.	Not applicable	User needs, preferences, and concerns; ideal use cases; desired features; and barriers to digital health adoption.
Knowledge database creation	Coconstruct and validate a user-informed chatbot knowledge base.	Coconstruction committee	Thematic analysis of workshops.	Content validation using the PEMAT^c^.	Curated questions and answers, key care decision-making points, and chatbot tone.
Prototyping and testing	Iteratively test and refine the CHAMP^d^ prototype.	Coconstruction committee	Thematic analysis of workshops, field notes, and observations.	Usability measures (eg, UMUX-Lite^e^ and AES^f^).	User experience outcomes (eg, tone, trustworthiness, and engagement), usability outcomes (eg, navigation, readability, responsiveness, and accessibility), and functional improvements per iteration.
Accessibility and multilingual adaptation	Identify and address barriers to CHAMP use among digitally excluded and multilingual users.	10 youth and 10 parents and caregivers overall	Thematic analysis of focus groups and interviews.	Not applicable	Accessibility needs, barriers, and solutions; translation issues; and adaptation recommendations.
Collaborative ethnography	Explore the process of coconstruction and its impact on CHAMP.	Coconstruction committee	Reflexive thematic analysis of observations, field notes, memos, interviews, and artifacts.	GRIPP2^g^ surveys	Thick description of the participatory process and impact on CHAMP development.

a AI: artificial intelligence.

b NASSS: Non-adoption, Abandonment, Scale-up, Spread, and Sustainability.

c PEMAT: Patient Education Materials Assessment Tool.

d CHAMP: CHatbot to Assist the Management of Pediatric patients.

e UMUX-Lite: Usability Metric for User Experience-Lite.

f AES: Acceptability E-Scale.

g GRIPP2: Guidance for Reporting Involvement of Patients and the Public.

#### Phase 1: Needs Assessment

##### Overview

A qualitative needs assessment will be conducted to identify the informational needs, preferences, and concerns of youth, parents, and caregivers regarding pediatric infections, AI, and chatbot use.

##### Approach

We will recruit 20 study participants (10 youths and 10 parents and caregivers). Four 2-hour focus groups [53], with 5 participants each, will be conducted. Focus group discussions will be guided by open-ended, semistructured questions exploring the ideal uses of health chatbots, desired content and functionalities, and anticipated benefits and concerns (Multimedia Appendix 1). To identify features associated with successful adoption, we will use the Non-adoption, Abandonment, Scale-up, Spread, and Sustainability framework [54]. This framework is frequently used to evaluate novel health technologies, focusing on the likelihood of adoptability, sustainability, and scalability.

##### Data Analysis

Focus groups will be audio-recorded, transcribed verbatim, deidentified, and analyzed using thematic analysis [55]. Findings will be cointerpreted with the coconstruction committee.

### Phase 2: Knowledge Database Creation

#### Overview

We will purposively invite a subset of Phase 1 participants to join the coconstruction committee, aiming for a diverse group of 12‐15 members. Together with the research team, the coconstruction committee will initiate and validate a comprehensive, user-informed knowledge database to serve as the foundation for CHAMP. This repository will feature an array of questions, appropriate responses, conversational frameworks, and simulated conversations.

#### Approach

Coresearchers will participate in 3-5 sequential workshops, every 2 hours in length, facilitated by members of the research team. Workshop activities will include (1) mapping key care questions and decision-making processes related to pediatric infections (eg, symptoms and when to seek medical care) and (2) providing feedback on chatbot response content (eg, clarity, tone, and accessibility). To improve the efficiency and validity of this process, we will leverage validated, open-access health resources published by professional organizations [42,43,56-58]. Health care providers on the research team will review all content to ensure medical accuracy and applicability to the local context.

#### Data Analysis

All workshops will be audio-recorded, transcribed verbatim, deidentified, and analyzed using thematic analysis to guide knowledge database refinement [55]. The validated Patient Education Materials Assessment Tool [59] will be used to evaluate the understandability and actionability of all materials included in the knowledge database. The engineering team will iteratively adapt the MARVIN architecture to integrate the validated knowledge database and create a preliminary CHAMP prototype.

### Phase 3: Iterative Prototyping and Testing

#### Overview

In this phase, the coconstruction committee will test the CHAMP prototype, providing feedback to guide the iterative refinement of its interface.

#### Approach

Coresearchers will participate in 3-5 sequential workshops, each lasting 2 hours. During these workshops, coresearchers will interact with the CHAMP prototype, engaging in live conversations to assess its performance and desired characteristics. Workshop activities will include (1) prototyping (evaluating CHAMP’s interface and structure at low fidelity) [60,61], (2) scenario testing (using hypothetical health scenarios to assess CHAMP’s functionality and decision logic) [62], and (3) the Wizard of Oz methodology (a simulated test where a human provides responses that users believe are automated to evaluate conversational flow, safety, and engagement) [63,64] (refer to Table 2). These activities will facilitate structured feedback on both preliminary usability (eg, navigation, responsiveness, and accessibility) and user experience (eg, tone, trustworthiness, and engagement) to identify optimal design solutions and interface options. Coresearchers will also complete validated measures of usability and acceptability after each workshop: the Usability Metric for User Experience-Lite [65] and the Acceptability E-Scale [66].

**Table 2.** Prototyping, scenario testing, and Wizard of Oz.

Activity	Definition	Rationale	Application in CHAMP^a^	Outcome
Prototyping	Creating a simplified, early version of the system to test design ideas.	Low development cost and flexible iterative changes; helps identify and fix early design flaws before testing real-world tasks.	Coresearchers will interact with an early CHAMP prototype (eg, mock-up or partial build) to evaluate layout, flow, and content structure.	Rapid feedback on interface and interaction design at low fidelity.
Scenario testing	Using realistic, hypothetical scenarios to assess how users interact with a system.	Builds on the prototype with realistic user behavior; reveals whether the prototype can support users in actual information-seeking situations.	Coresearchers will be given hypothetical pediatric health scenarios (eg, fever and cough) and asked to use CHAMP to seek guidance.	Test functionality and decision logic in meaningful health scenarios.
Wizard of Oz	A simulated test where a human provides responses that users believe are automated.	Simulates fully functional interactions before system development is complete; allows nuanced testing of tone, safety, trustworthiness, and engagement with chatbot responses before investing in full development.	Coresearchers will exchange short text-based messages for 10‐15 turns, and an HCP^b^ team member simulates CHAMP responses in real time.	Validate user experience and refine human-computer interaction details. This process ensures CHAMP can deliver accurate, easy-to-understand, and professional responses.

a CHAMP: CHatbot to Assist the Management of Pediatric patients.

b HCP: health care providers.

#### Data Analysis

All workshops will be audio-recorded, transcribed verbatim, and deidentified. Transcripts, field notes, and observational data from the workshop activities will be analyzed using a hybrid inductive and deductive approach, with coding structured around established usability dimensions while generating themes [55]. The usability and acceptability surveys will be analyzed using descriptive statistics (eg, mean, median, and SD). Qualitative and quantitative data will be triangulated to identify design features that enhance or hinder usability and acceptability.

Given the nature of development, Phases 2 and 3 will be a continuous, iterative process. Rapid feedback cycles between workshops will inform continuous prototype refinements. The engineering team will modify the prototype between workshops to integrate user feedback. This process minimizes the risk of poor chatbot performance in interpreting user input and ensures that CHAMP delivers responses that are clear, accessible, and appropriate for a lay audience. Iterative testing will continue until CHAMP reaches a satisfactory level of usability and acceptability, as determined by the research team.

### Phase 4: Accessibility and Multilingual Adaptation

#### Overview

This phase aims to further refine CHAMP and identify barriers to CHAMP adoption among populations at risk of digital exclusion.

#### Approach

We will conduct four 2-hour equity-oriented focus groups with a purposive sample of 20 participants (10 youths and 10 parents and caregivers), reflecting a range of digital barriers (eg, diverse gender, ethnocultural identities, socioeconomic status, immigration status, and digital literacy). Participants will be recruited through community organizations. Open-ended, semistructured questions will focus on perceived barriers to CHAMP use, targeting technical, cognitive, motivational, and accessibility challenges.

Next, to explore CHAMP’s multilingual functionality, we will integrate an automated translation layer into its core architecture, enabling real-time translation between English, French, Arabic, Spanish, Italian, and Mandarin [52]. Tool selection will be informed by an ongoing validation study, conducted in parallel by a graduate student on the research team, comparing multiple automated approaches, including LLM-based translation layers and established tools such as DeepL (DeepL SE) and Google Translate. Given the developmental scope of this phase, one professional translator per language will be contracted to evaluate translation quality through structured postinteraction interviews combining researcher-developed rating scales and open-ended questions. Translators will assess the following four dimensions: (1) accuracy and fidelity to source content, (2) clarity and readability at an approximate grade 6 level, (3) completeness, and (4) cultural appropriateness, with particular attention to safety-critical content such as red flag symptoms and emergency care instructions. Identified errors and concerns will be documented and escalated to the engineering team for iterative refinement before broader deployment and will inform safety monitoring protocols for future implementation phases.

#### Data Analysis

Focus groups and interviews will be audio-recorded, transcribed verbatim, deidentified, and analyzed using thematic analysis to identify barriers and solutions [55]. Categories and themes will be mapped across usability domains (eg, navigation, tone, clarity, and inclusivity) and language-specific issues. Translator rating scale data will be analyzed using descriptive statistics and triangulated with qualitative findings to identify patterns across languages and translation quality dimensions. The engineering team will aim to address the identified technical barriers. Crucially, the analysis will also attend to structural barriers that may not be resolved through engineering fixes alone (eg, distrust in digital health tools and exclusionary policies). In such cases, we will conduct additional focus groups with participants from equity-seeking or linguistically diverse populations to collaboratively identify feasible solutions for improved accessibility and multilingual considerations beyond technical refinements. The dual focus on both technical and structural dimensions of accessibility aims to ensure that CHAMP is both usable and inclusive in its design.

### Phase 5: Collaborative Ethnography

#### Overview

Throughout the study, we will explore the process of participatory coconstruction and its impact on CHAMP’s development through a focused collaborative ethnography [67,68]. This methodological approach emphasizes interdisciplinary collaboration at all stages of research and supports close examination of participatory health research [67]. The ethnography will focus on how coresearchers shape development and design decisions, how relational dynamics evolve within the research team, how values become embedded into CHAMP, and moments of consensus or tension in the negotiation of knowledge and technological features. This will enable examination of the process impacts of participation on coresearchers and CHAMP’s evolution.

#### Approach

Data collection will include (1) observations during workshops (Phases 2‐4), with detailed field notes documenting verbal and nonverbal interactions, group dynamics, and design negotiations; (2) reflexive memos written by coresearchers after each workshop; (3) semistructured interviews with coresearchers, exploring motivations, experiences, and perceived influence on CHAMP development; (4) five surveys capturing the GRIPP2 guidelines [40] to assess perceived role as coresearcher, level of engagement, costs, barriers, and facilitators to engagement; and (5) material artifacts, including the CHAMP prototypes, workshop outputs, chat histories, and email communications.

#### Data Analysis

Qualitative data (eg, field notes, memos, transcripts, and artifacts) will be analyzed using reflexive thematic analysis, drawing on analytical frameworks from participatory research [69]. Coding will attend to power asymmetries and negotiation dynamics, recognition of lived experience and expertise, and the emotional, relational, and practical dimensions of engagement, generating a thick description of the participatory process and impact on CHAMP development. Quantitative data from the surveys will be descriptively summarized and integrated with qualitative findings. This phase will help assess the transformative potential of participatory approaches in AI health research, including partner empowerment, mutual learning, and ethical commitments in practice.

### Sample Justification

The planned sample sizes reflect best practices for qualitative inquiry, participatory research, and pilot usability evaluation. The samples of Phase 1 (needs assessment) and Phase 4 (accessibility and multilingual adaptation), both of which involve focus groups with 20 participants, align with prior evidence that at least 3 focus groups with 5 participants capture 80% of themes and ensure acceptable data saturation [70]. For Phases 2 and 3, a cohort of 12‐15 coresearchers aligns with participatory design standards [71] and the minimum sample size recommended for pilot usability testing [72], promoting collaborative validation of chatbot content and function. Sustained engagement across phases ensures design continuity and depth of insight. In Phase 5, the ethnography prioritizes relational engagement, process dynamics, and lived experience, consistent with small-sample ethnographic traditions [68].

### Data Privacy and Security

During iterative prototyping and testing sessions, the following data will be collected for research purposes: prompts entered by coresearchers, chatbot responses generated by CHAMP, timestamps of interactions, and anonymized user codes. No personally identifiable information or personal health information will be required or expected to be collected.

All chat histories will be stored on secure servers hosted on Canadian cloud infrastructure (Amazon Web Services Canada Central), with backup storage on institutionally managed servers at the Research Institute of the McGill University Health Centre (MUHC). All data will be encrypted both at rest and in transit. Chat histories will be identified by a unique user code, and the key linking users to their assigned codes will be stored separately by the research team. Access will be restricted to study investigators and designated research team members involved in data analysis, as well as the MUHC Research Ethics Board (REB) for auditing purposes if required, through password-protected credentials. All team members will complete institutional privacy and REB training before accessing study data. Data will be stored for 7 years following study completion in accordance with MUHC REB policies, after which they will be securely destroyed.

Users will be instructed not to include any personal or identifiable information when interacting with CHAMP. As an additional safeguard, an automated filtering layer will screen all user inputs to flag and remove such information before processing by CHAMP. All interactions will be based on hypothetical scenarios, and chat histories will be reviewed by the research team to remove any inadvertently disclosed identifiable information before analysis. Deidentified chat histories may be used for iterative model refinement and improvement of CHAMP’s performance. Data will not be shared with external parties or used for any purpose beyond this study.

### Harms and Risk Management

Given the developmental nature of the study, performance errors may occur during iterative prototyping and testing, in which CHAMP provides inaccurate, unclear, or unusable information. We will explicitly communicate with coresearchers that CHAMP may make mistakes and is not yet a definitive source of health information. Identified errors will be documented and used to iteratively refine CHAMP.

Additional unintended harms, such as misunderstanding of chatbot content, discomfort, or safety concerns, will be captured through the workshops, surveys, and interviews. Any adverse events will be documented and reviewed by the principal investigator (EO). Investigators may pause or discontinue CHAMP use for an individual if there is evidence of distress, misuse, breach of confidentiality, or other safety concerns. Serious adverse events potentially related to CHAMP or study procedures will be reported to the REB. Oversight of safety, protocol adherence, and data integrity will be provided by the principal investigator, with external oversight and potential audits by the MUHC REB.

### Knowledge Dissemination

This study will use both integrated and end-of-grant knowledge translation. The coconstruction committee will be engaged throughout and inform key dissemination products. Study results will be shared with participants through plain-language summaries and with the public, health care providers, and other stakeholders through digital newsletters via community collaborators, social and traditional media, conference presentations, and peer-reviewed publications.

### Ethical Considerations

This protocol received approval from the MUHC REB on December 13, 2024 (MARVIN-CHAMP 2025‐10291), with an amendment for multilingual recruitment approved in June 2025. Informed consent will be collected from all participants before enrollment. Participation is voluntary, and participants can withdraw at any time with no consequences. All study data will be deidentified and stored securely in accordance with MUHC policies; participants will be informed of their rights regarding data withdrawal. Any important protocol modifications will be communicated to participants, coresearchers, and the MUHC REB through emails, team meetings, and formal amendment submissions.

## Results

This study is funded by the Unité de soutien au système de santé apprenant, Réseau universitaire intégré de santé et de services sociaux McGill, the Montreal Children’s Hospital MSSA Innovation Fund, and a Canadian Institutes of Health Research (CIHR) Operating Grant. The coconstruction committee is being assembled and includes 3 established partners and 5 newly recruited parent partners through the MUHC Partnership Office. As of December 2025, 4 initial meetings (held in February, March, April, and August 2025) have been completed. These meetings introduced CHAMP and gathered early insights on study design and data collection materials. They also generated input for the REB amendment and multilingual recruitment strategies. Participant recruitment for Phase 1 began in September 2025.

## Discussion

### Contribution to the MARVIN Master Protocol

This study operationalizes the MARVIN master protocol within a pediatric context [36], demonstrating how a flexible chatbot framework can be translated to new clinical populations. Grounding the CHAMP protocol within the master protocol provides a standardized and replicable development pathway, while allowing tailored adaptations (ie, coconstruction) for the needs of pediatric patients and families. Ultimately, this study will produce a functioning CHAMP prototype to support pediatric patients and families managing acute infectious symptoms. Findings will inform subsequent usability testing and a real-world implementation-effectiveness evaluation, while contributing to broader methodological and theoretical advancements in participatory and responsible AI development in pediatric health research [73].

### Anticipated Findings

The focus groups are expected to generate rich insights into the needs and preferences of youth and families managing pediatric infectious symptoms. They will identify key time points in the care journey where an AI chatbot such as CHAMP may be most beneficial, along with technical and structural considerations for adoption, including challenges related to the digital divide, linguistic accessibility, and trust in digital health tools.

The iterative workshops will inform both the conceptual foundations and practical design features of CHAMP, ensuring it is user-friendly, culturally sensitive, and effective in delivering validated health information. Collaboration between the coconstruction committee and the interdisciplinary research team will refine CHAMP’s content and features while guiding LLM prompt engineering. These participatory workshops aim to facilitate a dynamic feedback loop in which early prototypes are tested, critiqued, and improved in real time, enabling CHAMP to better meet users’ diverse needs and expectations. The iterative testing sessions are expected to yield valuable insights into CHAMP’s usability, acceptability, and perceived safety and will help identify early implementation considerations.

Finally, the collaborative ethnography will examine the internal dynamics of coconstruction during CHAMP’s development, opening the “black box” of how participatory approaches operate in practice [74]. This will deepen understanding of how coresearchers shape design decisions, how relational dynamics evolve, and how ethical values and lived experience become embedded into CHAMP. Studying these processes (eg, empowerment, trust-building, reciprocal learning, and negotiation of values) may address key gaps in participatory research and inform more transparent practices for child- and family-centered AI development.

### Study Limitations

Several limitations warrant consideration. The single-site design and small sample size across study phases may limit generalizability, although findings may still be transferable to other pediatric health contexts, particularly those seeking to develop equitable, user-informed AI health chatbots. Convenience and purposive sampling may introduce selection bias toward digitally engaged youth and families, potentially underrepresenting populations most affected by digital exclusion. While prototyping workshops are valuable for assessing early usability and acceptability, they can only approximate chatbot interactions and may not reflect real-world clinical use.

### Conclusion and Future Directions

This study emphasizes responsible AI and the importance of concomitant social innovation, enabled by participatory research and coconstruction. The next phases will involve a pilot usability test followed by an implementation-effectiveness study to evaluate real-world integration in pediatric care settings. Overall, CHAMP aims to transform the delivery of health care information for acute pediatric infectious symptoms. By providing timely, accurate, and accessible health information, CHAMP has the potential to reduce preventable ED visits, alleviate health care system burdens, and improve patient and family health care experiences.

## Supplementary material

10.2196/89852 Multimedia Appendix 1 CHAMP (CHatbot to Assist the Management of Pediatric patients) Phase 1 (needs assessment) focus group guide.

10.2196/89852 Checklist 1 SPIRIT-AI checklist.

10.2196/89852 Peer Review Report 1 Canadian Institutes of Health Research (CIHR) Operating Grant peer review report.

## Abbreviations

AI: artificial intelligence
CHAMP: CHatbot to Assist the Management of Pediatric patients
CIHR: Canadian Institutes of Health Research
ED: emergency department
GRIPP2: Guidance for Reporting Involvement of Patients and the Public
LLM: large language model
MARVIN: Minimal AntiRetroViral INterference
MUHC: McGill University Health Centre
REB: Research Ethics Board
SPIRIT-Al: Standard Protocol Items: Recommendations for Interventional Trials–Artificial Intelligence
