# Supplementary material for: Coconstructing CHAMP, an Artificial Intelligence Chatbot for Pediatric Infectious Symptoms Management: Protocol for a Multiphase Participatory Study
Source: JMIR Res Protoc. 2026 May 26;15:e89852. doi: 10.2196/89852 (PMC13205458; doi:10.2196/89852)
Supplement: Multimedia Appendix 1 [file resprot-v15-e89852-s001.pdf]

APPENDIX I

CHAMP Phase 1 Needs Assessment Focus Group Guide

Introduction

Hello. My name is [Moderator]. We would like to start off by thanking each of you for taking time to participate today. We will be here for about 2 hours. **The reason we are here today is to understand your experience accessing (online) pediatric health information and your perspectives about the role of a chatbot in supporting that**. We will be asking you questions and then encouraging and moderating a group discussion. We also would like you to know this focus group will be audio-recorded.

Guidelines for the Focus Group (Ground Rules)

To allow our conversation to flow more freely, we are going to review some guidelines:

1. Only one person speaks at a time. This is important as our goal is to make a written transcript of our conversation today.
2. Please avoid side conversations as this can be distracting.
3. Everyone does not have to answer every single question, but we'd like to hear from each of you today as the discussion progresses and so we may take time to make sure everyone has a chance to respond.
4. This is a confidential discussion in that we will not report your names or who said what to others. Names of participants will not be included in the final report that discusses the content of this meeting. It also means, except for the report that will be written, what is said in this room stays in this room.
5. We stress confidentiality because we want an open discussion. We want all of you to feel free to comment on each other's remarks without worrying that your comments will be repeated later and possibly taken out of context.
6. There are no "right" or "wrong" answers," just different perspectives. Please respect the opinions of others even if you do not agree with them.
7. Let me know if you need to pause for a break. You can stop the audio-recording at any time or remove certain excerpts of the recording. Participation is 100% voluntary.
8. Are there any questions before we get started?

(Start audio-recording)

Introduction of Participants

We would first like to go in a circle and have each of you introduce yourself. Please share your:

- Name
- Role as a patient or family member (i.e., I'm a father of a 13-year-old daughter, etc.)

Part 1: Access to Online Pediatric Health Information

1. How do you typically find health information for yourself [youth] or for your child [parent]?
    a. ***Where*** do you usually go to find pediatric health information?
        i. How comfortable are you using digital tools (apps, websites) to find health information?
    b. What ***kinds*** of pediatric health information do you usually look for?
    c. How do you decide if the information you find is ***trustworthy***?
    d. What are some ***challenges*** you face in finding or accessing pediatric health information? (language, readability, etc.)

Part 2: Perspectives on Chatbot Technology

2. What comes to mind when you hear the word "***chatbot***"?
3. Have you ***considered*** or ***used*** any chatbots (e.g., ChatGPT, Claude, Gemini) to find health information or make a health-related decision?
    a. ***Why*** did you decide to use a chatbot?
    b. What was that ***experience*** like?
4. [*Ideal uses and desired functionalities of health chatbots*]
    a. What ***roles*** could a chatbot play in pediatric healthcare?
        i. Probe: features, kinds of information
    b. What kinds of ***situations*** or ***healthcare settings*** could a chatbot help with?
        i. Probe: before/preventing ED visits, during ED visit, assisting post-ED discharge
    c. How should a chatbot ***deliver*** information or support?
        i. Probe: text, video, image, audio
5. [*Anticipated concerns*]
    a. Do you have any ***worries*** or ***concerns*** about chatbots in pediatric healthcare? (e.g., misinformation, bias, trust)
    b. What would make you ***trust*** the information provided by a health chatbot?
    c. What would make you ***stop trusting*** or ***decide not to use*** a health chatbot?
        i. Probe: around human/clinician oversight
    d. How should a chatbot handle ***sensitive topics*** or ***urgent health issues***?

Part 3: Wrap Up

6. What ***advice*** would you give researchers developing these technologies?
7. Is there ***anything else*** you would like to share about accessing pediatric health information or about health chatbots?
8. Thank you very much for answering our questions. Do you have ***any questions for us***?

End
