# Supplementary material for: Coconstructing CHAMP, an Artificial Intelligence Chatbot for Pediatric Infectious Symptoms Management: Protocol for a Multiphase Participatory Study
Source: JMIR Res Protoc. 2026 May 26;15:e89852. doi: 10.2196/89852 (PMC13205458; doi:10.2196/89852)
Supplement: Peer Review Report 1 [file resprot-v15-e89852-s003.pdf]

March/Mars 27, 2025

DC0190GP

Canadian Institutes of Health Research / Instituts de recherche en santé du Canada

Notice of Decision / Avis de décision

Application Number/Numéro de la demande: 529099
Committee Code/Code du comité: EHC

Applicants/Candidats: Dr. Esli Osmanlliu

With/Avec:				
	Professeur S. Achiche	Docteur S. Berthelot	Monsieur F. Bouajaj	Dr. L. Celi
	Dr. K. Engler	Dr. I. Gagnon	Dr. J. Gravel	Madame A. Grenier
	Mr. N. Jaiswal	Dr. L. Knisley	Docteur B. Lebouché	Docteur D. Lessard
	Monsieur P. Lewis	Ms. J. Lin	Mr. Y. MA	Ms. A. Ma
	Dr. D. Poenaru	Docteure M. Pomey	Prof. Z. Vang	

Institution paid/ Établissement payé: Research Institute of the McGill University Health Centre/Institut de recherche du Centre universitaire de santé McGill

Title/Titre: Co-designing an artificial intelligence-enabled CHatbot to Assist the Management of Pediatric patients (CHAMP) with common infections

Primary Inst./ Inst. principal: Human Development, Child and Youth Health / Développement et santé des enfants et des adolescents

Other Related Inst./ Autres inst. connexes:

**Competition Outcome/Résultats du concours:**	Operating Grant: ECRs in Human Development, Child and Youth Health / Sub. de fonct. : CDC en développement et la santé des enfants et adolescents<br>September/Septembre 24, 2024
**Number in competition/Nbre de demandes dans le concours:**	129
**Number approved in the competition / Nombre de demandes approuvées dans le concours:**	9

**Decision on your application/ Décision sur votre demande:**	Not Approved / Non approuvée
**Average annual amount/ Montant annuel moyen:**	$0

Peer Review Committee Recommendation, for your information and use/
Recommandation du comité d'examen par les pairs, pour fins d'information et d'utilisation:

**Committee/Comité:**	Early Career Researchers in Human Development, Child and Youth Health (2024) / Chercheurs en début de carrière en développ. et la santé des enfants et adolescents (2024)
**Application rank within the competition/ Rang de la demande dans ce concours:**	12
**Percent Rank Within the Competition/ Rang en pourcentage au sein du concours:**	9.3%
**Rating/ Cote:**	4.35
**Recommended average annual amount/ Montant annuel moyen recommandé:**	$50,000

Additional Funding Opportunities/ Opportunités de financement additionnelles	Decision/ Décision	Total Funding Amount/Montant total du financement	Term/ Terme	Competition Code/Cote de concours	Application Number/ Numéro de la demande
Op. Grant: ECRs in Human Dev., Child and Youth Health - Equity-Deserving Groups/Sub Fonct: CDC - dév. et santé des enfants et ado. - Groupes en quête d'équité	Approved/ Approuvée	$150,000	3 yrs/ans 0 mth/mois	202409EH8	543903

*** Applications receiving a score of less than 3.5 on any evaluation criteria will not be considered for Funding. / Les demandes qui ont reçu une note inférieure à 3.5 pour n'importe quel des critères d'évaluation ne sont pas admissibles.

This document is for information only. Official payment is stated on the CIHR Authorization for Funding.
Document à titre d'information seulement. Le paiement officiel est indiqué sur l'autorisation de financement des IRSC.

Review Type/Type d'évaluation: Committee Member 1/Membre de comité 1
Name of Applicant/Nom du chercheur: Osmanlliu, Esli
Application No./Numéro de demande: 529099
Agency/Agence: CIHR/IRSC
Competition/Concours: 2024-09-24 Operating Grant: Early Career Researchers in Human Development, Child and Youth Health/Subvention de fonctionnement : Chercheurs en début de carrière en développement et la santé des enfants et adolescents
Committee/Comité: Early Career Researchers in Human Development, Child and Youth Health (2024)/Chercheurs en début de carrière en développ. et la santé des enfants et adolescents (2024)
Title/Titre: Co-designing an artificial intelligence-enabled CHatbot to Assist the Management of Pediatric patients (CHAMP) with common infections

---

Assessment/Évaluation:

Summary:

Children with infectious symptoms (fever, cough, vomiting, diarrhea) frequently present to the emergency department and their families struggle to get trustworthy advice online or from health care providers, which may prevent the need for the visit. Questions from families are repetitive and predictable, thus amenable to automation. This study aims to use an artificial intelligence-enabled chatbot to assist management of pediatric patients (CHAMP) to support self-management from home, co-designed with families, healthcare providers and community organizations serving equity-deserving groups. Using large language models, chatbots can simulate human conversation and standardize information shared. Existing large language models lack family and community involvement in their development, may amplify health inequities due to barriers to digital access, overlook pediatric-specific requirements and use unverified sources of information.

Objectives and plan:

1- Engineering development of the chatbot in a mixed-methods study – Participants will include parents and teens who have visited an ED in the past 2 years, healthcare providers of pediatric patients, members of community organizations serving families from equity-desiring groups, Canadian ER physicians. Participant feedback will be iteratively incorporated into the adaptation of an existing successful chatbot (MARVIN, designed to help adults with HIV with medication self-management). Steps include: 1) needs assessment of ideal uses, functionalities and content, and anticipated concerns; 2) create a co-design committee with 12 -15 families and health care providers; 3) completion of design, development and testing of CHAMP with co-design committee through prototyping, scenario testing and simulated interactions; 4) conduct of 3-6 focus groups of 5 participants each , with purposive sampling among groups at risk of digital divide; 5) interpret results; 6) refine project’s knowledge mobilization strategy.

2- Determine usability and acceptability of the CHAMP prototype in a pilot implementation study at a Canadian pediatric emergency department serving a diverse patient population. 100 parents/caregivers and youth 14-17 years will interact with the chatbot over 3 weeks via phone or computer through a minimum of 20 written conversation turns (1 question and answer). Global Usability (Usability Metric for User Experience Lite as primary outcome, Health Education Impact Questionnaire)and acceptability testing (recruitment, retention) will be conducted using validated online survey instruments and 3 focus groups.

Strengths:

Diverse study team including both physicians and biomedical engineers, with engagement from families and community health care providers and organizations serving diverse and traditionally under-represented groups.

**Review Type/Type d'évaluation:**	Committee Member 1/Membre de comité 1
**Name of Applicant/Nom du chercheur:**	Osmanlliu, Esli
**Application No./Numéro de demande:**	529099
**Agency/Agence:**	CIHR/IRSC
**Competition/Concours:**	2024-09-24 Operating Grant: Early Career Researchers in Human Development, Child and Youth Health/Subvention de fonctionnement : Chercheurs en début de carrière en développement et la santé des enfants et adolescents
**Committee/Comité:**	Early Career Researchers in Human Development, Child and Youth Health (2024)/Chercheurs en début de carrière en développ. et la santé des enfants et adolescents (2024)
**Title/Titre:**	Co-designing an artificial intelligence-enabled CHatbot to Assist the Management of Pediatric patients (CHAMP) with common infections

Assessment/Évaluation:

Novel, technology-based solution to improve care and manage healthcare resources.

Significant pilot work already done and existing prototype will be adapted for this study.

Research plan is thoughtful, reasonable and clear.

KT plan is inclusive and thorough.

Weaknesses:
The study team will need to be very clear that the chatbot will not diagnose or perform triage of patients and is simply an informational tool, so it is not misused. It could be disastrous if a family felt they did not need to seek care based on information from the chatbot and their child had a bad health outcome.

Some frequent users of the emergency department may be children and families who speak languages other than French or English. They may also lack access to or skills to use digital technology. Although they may not be served by this pilot study, it will be important to engage them in the design phase and ensure that the chatbot can be adapted to be used with different technologies (laptop vs mobile phone vs landline, etc.) and ideally in other languages as well. These future considerations are alluded to but not full articulated in the proposal.

The study will test acceptability and usability of the chatbot, but no pilot data will be obtained to determine if this tool can prevent the need for an interaction with a healthcare provider.

Research Approach:
Sex and gender are considered in the analysis plan, as well as in the engagement of stakeholders and participants. Flexibility and attempts to remove barriers related to timing conflicts and transportation to allow participation is notable.

Applicant:
- Assistant Professor of Pediatrics, ER physician at Montreal Children’s Hospital/McGill University since 2019, Junior Clinician Scientist at RI-MUHC.

- FRSQ funding to provide salary support and has 50% protected research time.

**Review Type/Type d'évaluation:**	Committee Member 1/Membre de comité 1
**Name of Applicant/Nom du chercheur:**	Osmanlliu, Esli
**Application No./Numéro de demande:**	529099
**Agency/Agence:**	CIHR/IRSC
**Competition/Concours:**	2024-09-24 Operating Grant: Early Career Researchers in Human Development, Child and Youth Health/Subvention de fonctionnement : Chercheurs en début de carrière en développement et la santé des enfants et adolescents
**Committee/Comité:**	Early Career Researchers in Human Development, Child and Youth Health (2024)/Chercheurs en début de carrière en développ. et la santé des enfants et adolescents (2024)
**Title/Titre:**	Co-designing an artificial intelligence-enabled CHatbot to Assist the Management of Pediatric patients (CHAMP) with common infections

Assessment/Évaluation:

· Significant start-up funding for this project has already been obtained from various sources.

· Partnership with mentor from MIT with expertise in equitable deployment of artificial intelligence in health and with broad network of ER physicians to assist study as well as KT efforts through TREKK.

· 26 publications in last 5 years, 11 as first author

· ~$250 000 as PI of grants

Impact of Research:
Novel, technology-based solution to reduce low urgency emergency department health care service use in pediatric patients with common infections. If successful, could ultimately be used broadly to prevent unnecessary ER visits and serve as a credible source of pediatric health information. If successful, it could also be adapted to other pediatric and adult health conditions.

Quality of Research Environment: excellent
Budget:
High, but reasonable and is supplemented by other sources. It is possible that the research coordinator and research assistant roles will overlap, and the total FTE allocated could be slightly reduced.

Review Type/Type d'évaluation: Committee Member 3/Membre de comité 3
Name of Applicant/Nom du chercheur: Osmanlliu, Esli
Application No./Numéro de demande: 529099
Agency/Agence: CIHR/IRSC
Competition/Concours: 2024-09-24 Operating Grant: Early Career Researchers in Human Development, Child and Youth Health/Subvention de fonctionnement : Chercheurs en début de carrière en développement et la santé des enfants et adolescents
Committee/Comité: Early Career Researchers in Human Development, Child and Youth Health (2024)/Chercheurs en début de carrière en développ. et la santé des enfants et adolescents (2024)
Title/Titre: Co-designing an artificial intelligence-enabled CHatbot to Assist the Management of Pediatric patients (CHAMP) with common infections

---

Assessment/Évaluation:

Research approach

Summary: This project aims to develop a Chatbot (called CHAMP) to help respond to parents needs to manage their child's infections and avoid an unnecessary visit to the emergency dept (ED).The first study is to co-design the prototype, together with families and healthcare providers and the second study is to conduct a pilot implementation study in the ED to ensure its usability and acceptability of the prototype. 100 caregivers will participate and this will include youth <18 years . Participants will interact with CHAMP team over a 3-week period. Usability and acceptability will be surveyed. The goal is to avoid visits to ED that are not necessary and to provide accessible, timely and trustworthy evidence-based bilingual information to users. A strength is the involvement of the user, parents of sick children in the co-design of the chatbot, with consideration of <u>equity deserving groups</u> needs. This will ensure the chatbot meets the knowledge needs of parents of young children with signs of infection (i.e. should I bring my child to ED? If not, what care should I provide?) and that it is user friendly. It is also important that knowledge tools are tailored for pediatric health care (as opposed to adults). The developed tool will be implemented by parents/caregivers in the ED. What is not clear; is this meant for parents to aid in decision making <u>before</u> coming to ED or once they are in the waiting room of ED. Acceptable measures will assess usability, acceptability and impact. It is important to develop it in Canada with the Canadian health care context considered. Healthcare providers will assist in developing content; eg what are the expected questions and what is the health decision re management. This research group will benefit from lessons learned of the local experiences of another chatbot developed (MARVIN, for adults with HIV). Co-design will focus on usability and accessibility. Outcome is the successful development of the CHAMP and that it is implementable (i. e. usable , acceptable).This project will include <u>equity deserving</u> groups in their patient partners (PWLE) who will be at higher need for information when making a decision of whether or not to go to the ED with their child who has signs of infection (specifically; low SES, single-parent households, gender diverse, language barriers , newcomers to Canada, low literacy levels, rural). This diversity is ensured by collaborations with PROMIS, Garage a Musique, and other community organizations that support equity deserving groups of families. The co-design committee for CHAMP is also selected based on diversity. This is a strength of this proposal.

- **The MARVIN was developed for adults with health knowledge needs. Are you anticipating any content and format issues that may be different for caregivers of children when compared to adult patients with HIV (different targets)?**

- **It would have been helpful to give examples of what content would include to appreciate impact.**

**Review Type/Type d'évaluation:**	Committee Member 3/Membre de comité 3
**Name of Applicant/Nom du chercheur:**	Osmanlliu, Esli
**Application No./Numéro de demande:**	529099
**Agency/Agence:**	CIHR/IRSC
**Competition/Concours:**	2024-09-24 Operating Grant: Early Career Researchers in Human Development, Child and Youth Health/Subvention de fonctionnement : Chercheurs en début de carrière en développement et la santé des enfants et adolescents
**Committee/Comité:**	Early Career Researchers in Human Development, Child and Youth Health (2024)/Chercheurs en début de carrière en développ. et la santé des enfants et adolescents (2024)
**Title/Titre:**	Co-designing an artificial intelligence-enabled CHatbot to Assist the Management of Pediatric patients (CHAMP) with common infections

---

Assessment/Évaluation:

· My understanding is that the chatbot is meant to be used by caregivers before going to ED , to avoid unnecessary visits. Why is the implementation study not targeting these users?

· What is a challenge of all applications is sustainability of eHealth tools and I wonder if there is a sustainability plan (e.g. to continually update the chatbot to align with continuous software upgrades of smartphones and laptops, changes in practice).

· For the implementation trial: it would be of value to interview a subset of participants (i.e. not only self-report measures) to get specific info about their user experience and how useful it was for their (diversity) context.

· It is interesting (=important, may impact appropriateness) that you will measure eHealth literacy as a possible barrier to accessibility and adoption.

· Sample size justification is provided. Can you clarify what you mean by “groups at risk for digital exclusion” (give examples)

· There is a KT plan but the target of the efforts is not clear. Most proposed KT plans are targetting health professionals. What is the dissemination plan, i.e. to get in the hands of parents of young children. How will parents be made aware of the chatbot?

Originality of the Proposal
Chatbots are gaining popularity with AI and machine learning that enables health questions posed by families to receive evidence based responses, which can save healthcare costs.

Applicant
The applicant is a pediatrician (emergentologist) and has completed a MSc in Epidemiology at McGill University. He has several awards for leadership and social justice in medicine. He has a Clinician Scientist Scholar award funded by FRQS (2023-27,Junior 1)and receives infrastructure support from the Research Institute of the McGill University Health Centre (e.g. IT, space for meetings, statistical support, contracts, office space, etc). He is on the Steering committee of *Children’s Journey* (a hospital

**Review Type/Type d'évaluation:**	Committee Member 3/Membre de comité 3
**Name of Applicant/Nom du chercheur:**	Osmanlliu, Esli
**Application No./Numéro de demande:**	529099
**Agency/Agence:**	CIHR/IRSC
**Competition/Concours:**	2024-09-24 Operating Grant: Early Career Researchers in Human Development, Child and Youth Health/Subvention de fonctionnement : Chercheurs en début de carrière en développement et la santé des enfants et adolescents
**Committee/Comité:**	Early Career Researchers in Human Development, Child and Youth Health (2024)/Chercheurs en début de carrière en développ. et la santé des enfants et adolescents (2024)
**Title/Titre:**	Co-designing an artificial intelligence-enabled CHatbot to Assist the Management of Pediatric patients (CHAMP) with common infections

Assessment/Évaluation:

committee of the Montreal Children's Hospital [MCH]) that leverages digitial health innovation to enhance patient care (important for KM). He is the MCH representative on Pediatric Emergency Research Canada (PERC; national). He started his academic career 2019 at the ED- MCH.

His research program focuses on telemedicine and digital health; areas that are very important in delivering health care to those who live far from specialty care (rural).

Publications: 26 publications, 25 since his appointment

Funds: He is PI on competitive funds to include a Royal College travelling fellowship, an MSSA Innovation Fund Competition, and a Systeme de sante Apprenant Quebec and SGB Kids Grant.

Supervision: 4 Undergrads, 1MSc thesis , 1 MSc non thesis, 1 PhD. 2 certificate

Impact of the Research
This will have an impact on parents of sick children with infections by empowering them to know if they need to seek medical support of an emergency room in acute care. This is potentially cost-effective since the number of visits to the ED can be diminished by unnecessary visits. Child health can be improved as parents are provided with the knowledge on how to deal with their child's infection successfully. Equity deserving groups will have access to a user friendly chatbot that meets their literacy level and knowledge needs.

Quality of the Research Environment
A large(>25) impressive interdisciplinary team (roles and expertise clarified) has been assembled to support this project. There are engineers who will adapt MARVIN (i.e. an existing chatbot) to CHAMP. This will greatly expedite development. Parent partners are engaged to co-design the chatbot; there will be a needs assessment (focus group) to discuss with caregivers and health professionals what the knowledge gaps are. This will inform content and format of the chatbot. There are relevant community organizations involved and research networks which will help with recruitment of a diversity of participants and also dissemination. Diversity (gender and ethnicity, sociodemographic profile) of participants is sought after.

The applicant is supported by the Research Institute of the MUHC.

**Review Type/Type d'évaluation:**	Committee Member 3/Membre de comité 3
**Name of Applicant/Nom du chercheur:**	Osmanlliu, Esli
**Application No./Numéro de demande:**	529099
**Agency/Agence:**	CIHR/IRSC
**Competition/Concours:**	2024-09-24 Operating Grant: Early Career Researchers in Human Development, Child and Youth Health/Subvention de fonctionnement : Chercheurs en début de carrière en développement et la santé des enfants et adolescents
**Committee/Comité:**	Early Career Researchers in Human Development, Child and Youth Health (2024)/Chercheurs en début de carrière en développ. et la santé des enfants et adolescents (2024)
**Title/Titre:**	Co-designing an artificial intelligence-enabled CHatbot to Assist the Management of Pediatric patients (CHAMP) with common infections

Assessment/Évaluation:

Comments on the budget

The applicant has used some internal funds to start development of the CHAMP. There is some overlap with existing grants (MSSA funding). Budget requested will be for a research coordinator and research assistant, PhD funding, engineers, summer students, web services, recruitment materials and activities, consultation fees, compensation for Patient partners using CHILD-BRIGHT and CEPPP guidelines, publication costs, travel for trainees to conferences, knowledge brokers. All appears appropriate.

Integration of Sex and/or Gender in the Research Proposal

Diversity of involvement of participants in the co-design , to include sex and gender. Will consider the unique barriers associated with gender for participation (caregiver responsibilities) and will try and mitigate these barriers. Flexibility for meeting schedules will be implemented to ensure broad participation. Sex and gender will be considered in analysis of outcomes of CHAMP in the ED contexts.
